# Comparing the effects of mHealth app use and face-to-face training on the clinical and laboratory parameters of dietary and fluid intake adherence in hemodialysis patients: a randomized clinical trial

**DOI:** 10.1186/s12882-023-03246-7

**Published:** 2023-06-29

**Authors:** Mohsen Torabikhah, Zahra Farsi, Seyedeh Azam Sajadi

**Affiliations:** 1 Medical-Surgical Department, Nursing School, Aja University of Medical Sciences, Tehran, Iran; 2 Research and Community Health Departments, Nursing School, Aja University of Medical Sciences, Tehran, Iran; 3 Nursing Management Department, Nursing School, Aja University of Medical Sciences, Tehran, Iran

**Keywords:** Telemedicine, mHealth, Education, Treatment adherence, Hemodialysis

## Abstract

**Background:**

Numerous factors are likely to result in poor treatment adherence, which is one of the important factors contributing to increased complications and the low efficacy of hemodialysis (HD), particularly inadequate knowledge of patients. This study aimed to compare the effects of a mobile health (mHealth) app (the Di Care app) use and face-to-face training on the clinical and laboratory parameters of dietary and fluid intake adherence in patients undergoing HD.

**Methods:**

This single-blinded, two-stage/two-group randomized clinical trial was fulfilled in 2021-22 in Iran. Seventy HD patients were recruited, using the convenience sampling method, and were then randomized into two groups: mHealth (n = 35) and face-to-face training (n = 35). ​ The patients in both groups received the same educational materials via the Di Care app and face-to-face training for one month. Before and 12 weeks after the intervention, the mean interdialytic weight gain (IDWG), potassium (K), phosphorus (P), total cholesterol (TC), triglyceride (TG), albumin (AL), and ferritin (FER) levels were measured and compared. The data were analyzed using the SPSS via descriptive statistics (mean, SD, frequency, and percentage) and analytical tests (independent-samples *t*-test, paired-samples *t*-test, Wilcoxon signed-rank test, Mann-Whitney U test, Chi-square test, and Fisher’s exact test).

**Results:**

​Prior to the intervention, the mean IDWG and the K, P, TC, TG, AL, and FER levels, were not significantly different in both groups (*p* > 0.05). The mean IDWG (*p* < 0.0001), and the K (*p* = 0.001), P (*p* = 0.003), TC/TG (*p* < 0.0001), and FER (*p* = 0.038) levels in the HD patients in the mHealth group decreased. ​As well, the mean IDWG (*p* < 0.0001), and the K (*p* < 0.0001) and AL (*p* < 0.0001) levels showed a descending trend in the face-to-face group. The fall in the mean IDWG (*p* = 0.001) and the TG level (*p* = 0.034) in the patients in the mHealth group was significantly greater than that in the face-to-face group.

**Conclusions:**

The Di Care app use and the face-to-face training could improve dietary and fluid intake adherence in patients. However, mHealth could have more effect on the laboratory parameters than face-to-face training, largely reducing the IDWG.

**Trial registration:**

This study was registered in the Iranian Registry of Clinical Trials (No. ID IRCT20171216037895N5).

## Background

Chronic renal failure (CRF) has been declared the 12th leading cause of death worldwide [1]. The mortality rate due to this condition has also surged by 41.5% from 1990 to 2017, while the death rates following cardiovascular diseases and types of cancer have fallen by 30.4% and 14.9%, respectively, in the same period [2].

When CRF progresses and patients reach the end stage of kidney disease (ESKD), renal replacement therapy (RRT), including hemodialysis (HD) as the most common treatment modality [3], is required [4]. Of note, over 29,000 patients in Iran have been undergoing HD in 2016 [5].

However, adequate healthcare services are rarely delivered to ESRD patients in low- and middle-income countries, including Iran. For instance, in Asia, 17–34% of people need to take advantage of RRT [6]. Although some of them do not have access to this therapy because of problems such as high costs of treatment and besides, others, who have access, experience high rates of various mental and physical complications, e.g., depression, anxiety, and hypertension [3, 7].

Treatment non-adherence is one of the significant factors contributing to increased complications and the low efficacy of HD [8, 9]. In this line, some studies have reported poor treatment adherence among patients undergoing HD [10–13], which can cause weight gain, elevated serum phosphorus (P) and potassium (K) levels, decreased albumin (AL), andimpaired fat profiles [14–16]. It seems that among the dimensions of treatment adherence, adherence to dietary recommendations and fluid intake restrictions are needed to be considered more [17]. Failure to comply with nutritional guidelines and fluid intake limitations by these patients can thus cause malnutrition adequacy [18].

Numerous factors are likely to result in poor treatment adherence, particularly inadequate knowledge [10–12, 19], which was considered as one of the most important factors that affect adherence to treatment besides other variables like attitude and satisfaction [20]. Various training methods have been so far implemented to provide the required information in this respect. Previous studies have suggested traditional training methods along with novel technologies, such as mobile apps [21–23]. Many studies have further investigated the effects of traditional training methods, such as face-to-face training and educational pamphlets or newer methods, i.e., the use of the Short Message Service (SMS) and telephone follow-up (TFU) on treatment adherence in HD patients [24–26]. However, the authors did not find a study regarding the effects of mHealth app use on the clinical and laboratory parameters of dietary and fluid intake adherence and comparing it with one of the traditional training methods. Therefore, this study aimed to design and build a mHealth app for HD patients and compare the effects of using this app and face-to-face training on the clinical and laboratory parameters of dietary and fluid intake adherence in HD patients.

## Methods

### Design

This study was part of a single-blind, two-stage/two-group randomized clinical trial fulfilled in 2021-22 in one of the main HD centers in Isfahan, Iran, and registered in the Iranian Registry of Clinical Trials (No. ID IRCT20171216037895N5, Date: 28/04/2021). Before designing this study, a structural search was done and after investigating related published articles, the present method was designed. Studies from 2010 to 2022, which were published in PubMed, Science Direct, Google Scholar, Magiran, and SID, were included and education, patient education, face-to-face, mobile applications, mobile Health (mHealth), adherence, and adherence to treatment were used as keywords in the mentioned search.

### Participants and sample size

The sample size was calculated with 95% confidence interval and 80% test power, based on the standard deviation (SD) of treatment adherence to 4.9, obtained from the previous similar study [24] and using the sample size formula below:

$$\displaylines{n = \frac{{{{(2\sigma )}^2}{{\left( {{Z_{1 - \alpha /2}} + {Z_{1 - \beta }}} \right)}^2}}}{{{d^2}}} = \cr \frac{{{{(2 \times 4.90)}^2}{{\left( {1.96 + 0.84} \right)}^2}}}{{{{(0.7 \times 4.90)}^2}}} = 64 \cr}$$

Then, considering the 10% sample loss in each group, 35 individuals were calculated. The inclusion criteria were the patients’ willingness to​ participate in the study, aged over 18, the ability to read and write, access to a smartphone with an Android operating system, undergoing HD within the study setting permanently, experiencing three HD sessions per week, receiving HD for at least six months, using no other mHealth apps along with HD during the study, no mental illnesses or other chronic diseases, such as heart failure, and no disability causing disorders in the training process. In addition, unwillingness to continue cooperation with the study, being transferred to other centers, undergoing a kidney transplant during the study, not using the Di Care app for more than one week, not attending the face-to-face training sessions for ​over three sessions, and facing inadequate functioning of the Di Care app on smartphones were the exclusion criteria.

The HD patients were recruited in this study using convenience sampling method by one of the researchers. All patients, who were in the center, enrolled in the study. Then, among those who were eager to participate, other criteria were checked to reach the calculated sample size. The study objectives were then explained to them, and informed consent was obtained. Afterward, the patients were randomized into two groups: mHealth (n = 35) and face-to-face training (n = 35) via tossing a coin, so the coin was tossed for each eligible patient and if the patients were on the coin head, they entered into the face-to-face training group, and if they were on the coin tail, they were included in the mHealth app use one. This procedure continued until the desired sample size was reached. To prevent the exchange of information between the patients in both groups, besides asking the patients not to exchange information until the end of the study, the app server was controlled daily during the intervention for the registration of patients in the face-to-face training group.

### Data collection

To evaluate the effects of the interventions on treatment adherence among HD patients, the mean IDWG and the laboratory parameters were measured. One week before the intervention, the mean IDWG was recorded using the Seca 676 medical scale. To evaluate the reliability of the scale before recording the patients’ weight, the weight of five people in the same gown was measured twice at one-minute intervals, whose correlation coefficient was equal to 0.9. Weight measurement was also performed before undergoing HD and in hospital gowns. To prevent bias, it was practiced and supervised by a researcher and two trained nurse assistants unaware of patient groups. In the first training session, immediately after the patients were connected to the HD machines, 5 ml blood samples were taken from an arterial catheter to measure the serum K, P, total cholesterol (TC), triglyceride (TG), AL, and ferritin (FER) levels. In order to check K, Easy Electrolyte® kit and device were used. TG, AL, FER, and P level was measured by enzymatic method and Pars Azmoon® kit, bromocresol green test and Pars Azmoon® kit, ELISA method and Pishtaz Teb® kit, and UV test and Pars Azmoon® kit respectively. All biochemistry test was done by Hitachi® model 902. All the experiments were performed with laboratory devices and kits, and the same measurement methods in the study setting. All the tests were completed by a laboratory technician unaware of patient groups.

### Intervention

In the mHealth group, the patients utilized a researcher-made mobile-based app on the Android platform. This app is being registered under the Di Care trademark in the Intellectual Property Center of the Islamic Republic of Iran, whose main language was Persian. Before designing the app, patients’ needs and other similar applications and studies were investigated and checked by the researchers, and the final version was published after a long-term revision, which was held by the researchers and application developers. In the first training session, the Di Care app was installed on the patients’ smartphones in the mHealth group and its correct operation was ensured. For example, a reminder was set for one of the patient’s medications. To confirm the correct operation of the software, ​one of the researchers, with a master’s degree in nursing and two years of experience in the HD center, met the patients in the group trained with the app weekly and answered their possible questions.

**Fig. 1:**
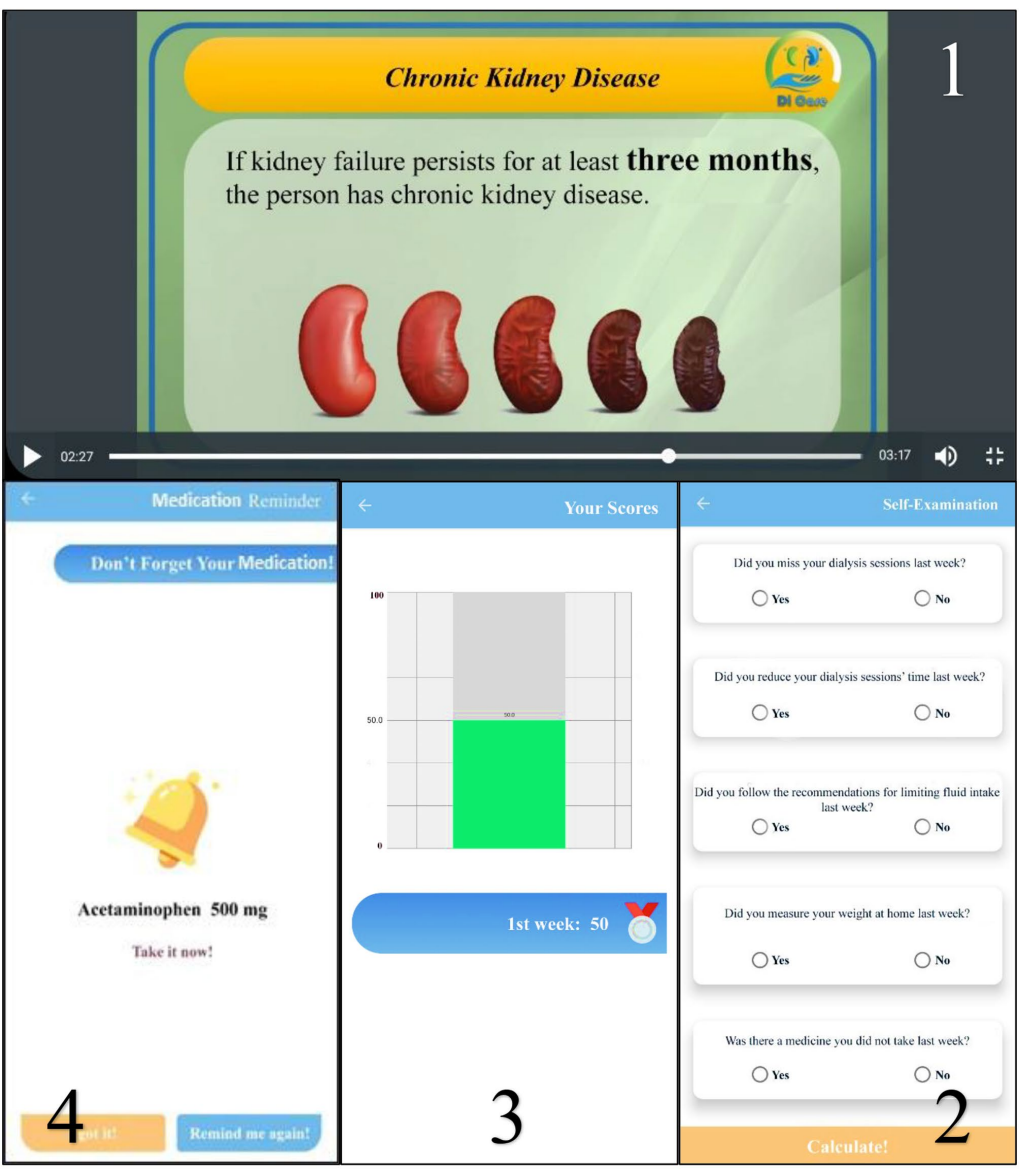
Different parts of Di Care app. **(1)** Using image, text and audio simultaneously in Di Care app **(2)** Weekly self-test **(3)** Comparison of scores obtained in different weeks **(4)** Medication reminder

The Di Care app consisted of different features. Its educational materials were prepared in three-minute videos on seven topics for the patients. In these videos, some images, texts, and audios were used simultaneously (Fig. 1). The topics were mainly about the importance of HD, the management of HD complications, diet, fluid intake restrictions, physical activities, vascular access care, and medications. The educational content, which was published finally as a book named “Hemodialysis Care for Patients”, was gathered from updated guidelines and pieces of literature. When the final version was proven by the Authoring and Translation Committee of Aja University of Medical Sciences, Tehran, Iran, the researchers started creating videos based on the mentioned topics. Three notifications could be also sent to the patients offline at certain times of the day. By touching the notifications, the users would see one of the educational videos uploaded to the app server. Three videos with different topics could be further shown daily to the patients with no repetition for one month. The app could intelligently show the users the notification to participate in the self-test every seven days from the time of launching on the smartphone. By touching this message, the users could participate in a self-test offline to assess the level of adherence to the recommendations provided (Fig. 1). The 10 questions in this self-test were adapted from the End Stage Renal Disease Adherence Questionnaire (ESRD-AQ). The users even viewed the scores and compare them by week. The Di Care app could also make it possible to record medications for reminders, and the patients benefited from them (Fig. 1). Some information, such as foods high in sodium (Na) and K and the amount and type of physical activity was always available on a table to the users. The information recorded in the software, including the scores obtained, the videos viewed, the most viewed videos, and the number of days of using the software, could be also sent to the server and stored in the users’ account, allowing researchers to check their status in absentia.

In the face-to-face training group, the educational materials prepared for the Di Care app were taught to the patients in person by the same researcher who evaluated the correct functioning of the software. The patients were thus consulted about the timing of the training during HD. The training was completed in 12 sessions (for four weeks and three sessions per week) and lasted for at least 10 min. The training sequence was the same as that provided by the app. At the end of each training session, the patients could also raise their questions about the issues related to the topics taught. In addition, at the end of each session per week, the educational materials taught in the previous week were delivered in writing to the patients.

**Fig. 2:**
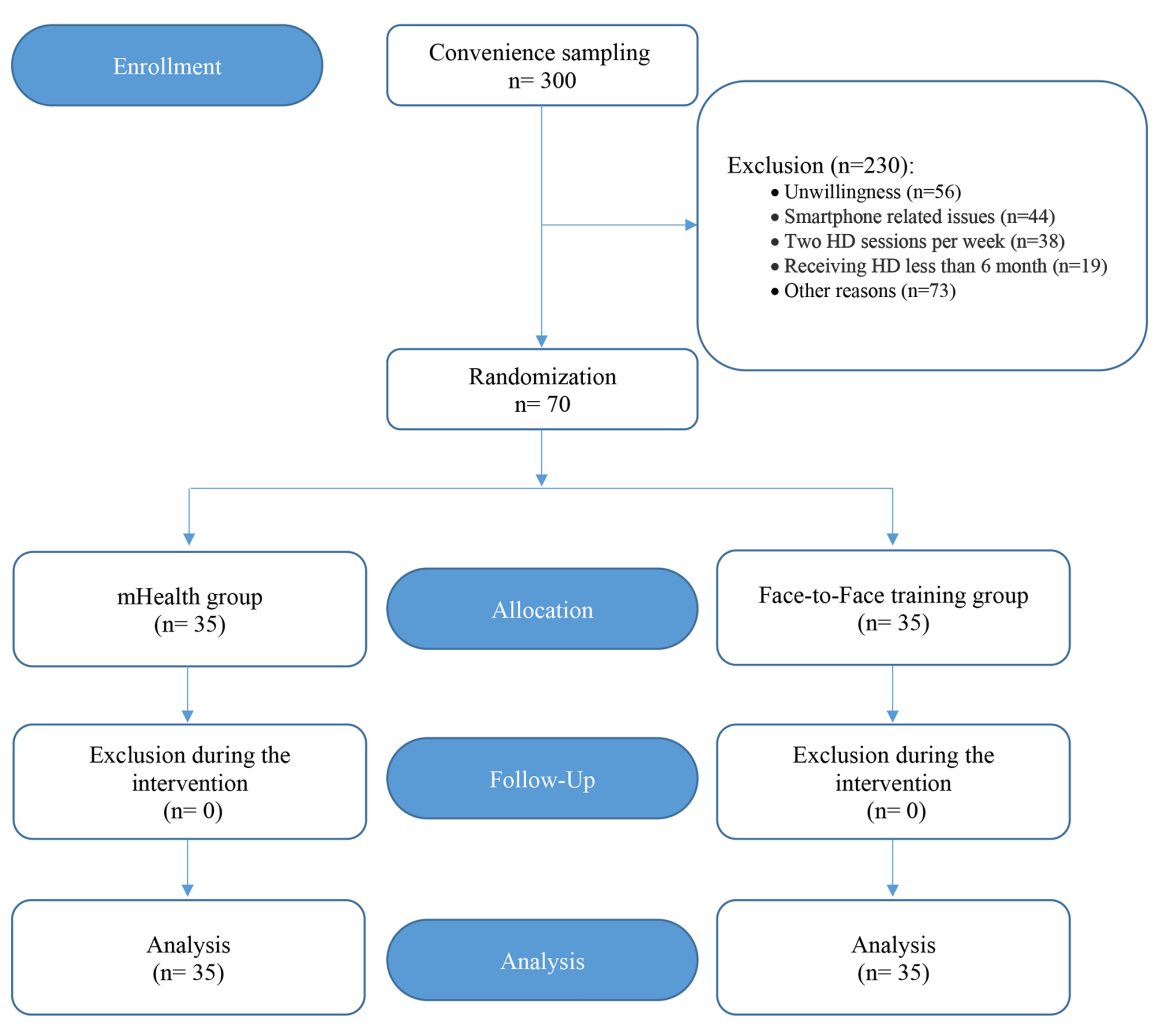
The study process

​Twelve weeks after the onset of the intervention program (viz. eight weeks after the last training session), the mean IDWG in the patients was measured and recorded in the three training sessions of the twelfth week, the blood samples were taken to measure the serum K, P, TC, TG, AL, and FER levels, and sent to the hospital laboratory, where the study was being performed. The criteria for dietary and fluid intake non-adherence were as following: the IDWG of more than 5.7% of dry weight, the serum K level of more than 6 mmol/l, P of more than 7.5 mg/dl, TC of more than 200 mg/dl, TG of more than 150 mg/dl, AL less than 3.5 g/dl, and FER less than 30 and more than 300 µg/l [14, 16, 27–29]. The study process is displayed in Fig. 2.

### Data analysis

The data were analyzed using the SPSS Statistics software (ver. 16) via Kolmogorov-Smirnov test to checking normality, descriptive statistics (i.e., mean, SD, frequency, and percentage), and analytical tests (that is, independent-samples *t*-test, paired-samples *t*-test, Wilcoxon signed-rank test, Mann-Whitney U test, Chi-square test, and Fisher’s exact test). The significance level was also considered by *p* < 0.05. The data was analyzed by a biostatistician, unaware of patient groups and interventions.

## Results

### Characteristics of patients

The mean age of the patients was 46 ± 8.84 (20–60 years) with an average of 3.34 ± 1.26 (1–6 years) HD. 49% of the patients were male. Both study groups were not significantly different in terms of age (*p* = 0.486), gender (*p* = 0.794), duration of HD (*p* = 0.341), dry weight (*p* = 0.108), type of vascular access (*p* = 0.738), and level of education (*p* = 0.476) (Table 1).

**Table 1:** Comparison of individual characteristics of patients in the two groups

Variable		Group		Statistical value		
		mHealth	Face-to-Face	t	df	*p*-value
		Mean (SD)	Mean (SD)			
**Age (year)** ^*^		45.2 (1.4)	46.7 (1.5)	0.70	68	0.486
**Duration of hemodialysis treatment (year)** ^*^		3.2 (0.2)	3.4 (0.2)	-	-	0.341
**Dry weight (Kg)** ^*^		71.6 (2.1)	66.5 (2.2)	1.62	68	0.108
**Variable**		**f (%)**	**f (%)**	**χ** ^**2**^	**df**	***p*** **-value**
**Sex** ^**^	**Male**	24 (49.0)	25 (51.0)	1	1	0.794
	**Female**	11 (52.4)	10 (47.6)			
**Vascular access** ^***^	**Arterial-Venous fistula**	13 (56.5)	10 (43.5)	-	2	0.738
	**Arterial-Venous graft**	4 (44.4)	5 (55.6)			
	**Catheter**	18 (47.4)	20 (52.6)			
**Education** ^***^	**Elementary**	6 (17.1)	10 (28.5)	-	3	0.476
	**Guidance**	11 (31.4)	12 (34.2)			
	**High school**	13 (37.1)	11 (31.4)			
	**Academic**	5 (14.2)	2 (5.7)			

SD: Standard Deviation, df; degree of freedom, f: frequency

^*^ Independent *t*-Test, ^**^ Fisher’s exact test, ^***^ Chi-square test

### Primary outcomes

The independent-samples *t*-test revealed that the patients of the two groups were not significantly different before the intervention regarding mean IDWG (*p* = 0.785), the serum K (*p* = 0.243), P (*p* = 0.370), TC (*p* = 0.803), TG (*p* = 0.905), AL (*p* = 0.087), and FER (*p* = 0.756) levels (Table 2). In addition, no significant difference was observed after the intervention in both groups in terms of the mean serum K (*p* = 0.349), P (*p* = 0.441), TC (*p* = 0.642), AL (*p* = 0.932), and FER (p = 0.545) levels. However, the mean IDWG (*p* = 0.001) and the serum TG (*p* = 0.034) level in the mHealth group was significantly lower than that in the face-to-face training one (Table 2).

**Table 2:** Comparison of clinical and laboratory parameters before and after the intervention in the two groups

Intergroup comparison						Intragroup comparison		
Variable	Stage	Group	Mean (SD)	Independent *t*-Test		Group	Paired *t*-Test	
				t^*^	*p*-value		t^**^	*p*-value
**IDWG** **(Kg)**	Before	mHealth	3.6 (0.8)	-0.27	0.785	mHealth	-8.05	< 0.0001
		Face-to-Face	3.7 (0.7)					
	After	mHealth	2.3 (0.7)	-3.45	0.001	Face-to-Face	-6.023	< 0.0001
		Face-to-Face	2.9 (0.6)					
**K** **(mmol/l)**	Before	mHealth	5.1 (0.4)	1.17	0.243	mHealth	-3.78	0.001
		Face-to-Face	5.0 (0.3)					
	After	mHealth	4.6 (0.6)	0.94	0.349	Face-to-Face	-4.83	< 0.0001
		Face-to-Face	4.4 (0.5)					
**P** **(mg/dl)**	Before	mHealth	4.3 (0.9)	0.90	0.370	mHealth	-3.21	0.003
		Face-to-Face	4.1 (0.9)					
	After	mHealth	3.6 (0.6)	-0.77	0.441	Face-to-Face	-1.70	0.097
		Face-to-Face	3.7 (0.6)					
**TC** **(mg/dl)**	Before	mHealth	203.2 (44.1)	-0.25	0.803	mHealth	-4.22	< 0.0001
		Face-to-Face	205.8 (41.5)					
	After	mHealth	158.3 (36.3)	-0.46	0.642	Face-to-Face	-4.83	< 0.0001
		Face-to-Face	162.5 (39.3)					
**TG** **(mg/dl)**	Before	mHealth	213.9 (114.1)	0.12	0.905	mHealth	-4.36	< 0.0001
		Face-to-Face	210.7 (106.4)					
	After	mHealth	155.4 (76.1)	-2.16	0.034	Face-to-Face	-0.91	0.365
		Face-to-Face	198.8 (90.8)					
**AL** **(g/dl)**	Before	mHealth	3.7 (0.4)	1.73	0.087	mHealth	1.91	0.064
		Face-to-Face	3.5 (0.4)					
	After	mHealth	3.9 (0.2)	-0.08	0.932	Face-to-Face	3.88	< 0.0001
		Face-to-Face	3.9 (0.2)					
**FER** **(µg/l)**	Before	mHealth	289.7 (181.6)	0.31	0.756	mHealth	-2.15	0.038
		Face-to-Face	275.0 (211.9)					
	After	mHealth	207.8 (99.2)	-0.60	0.545	Face-to-Face	-1.430	0.162
		Face-to-Face	221.7 (91.2)					

SD: Standard Deviation, df; degree of freedom, IDWG: interdialytic weight gain, K: potassium, P: phosphorus, TC: total cholesterol, TG: triglyceride, AL: albumin, FER: ferritin

^*^df = 68, ^**^df = 34

**Table 3:** Non-adherence to diet and fluid intake before and after the intervention in the two groups

**Intergroup comparison**							**Intragroup comparison**			
**Variable**	**Non-adherence criteria**	**Stage**	**Group**	**f (%)**	**Mann-Whitney U**		**Group**	**Wilcoxon Signed Ranks**		
					**Z**	***p***		**Z**	***p***	
**IDWG** **(Kg)**	> 5.7% of dry weight	Before	mHealth	13 (37.1)	-1.89	0.058	mHealth	-3.60	< 0.0001	
			Face-to-Face	21 (60.0)						
		After	mHealth	0 (0.0)	-2.98	0.003	Face-to-Face	-3.60	< 0.0001	
			Face-to-Face	8 (22.8)						
**K** **(mmol/l)**	> 6	Before	mHealth	2 (5.7)	-1.42	0.154	mHealth	-1.41	0.157	
			Face-to-Face	0 (0.0)						
		After	mHealth	0 (0.0)	0.00	1.00	Face-to-Face	0.00	1.00	
			Face-to-Face	0 (0.0)						
**TC** **(mg/dl)**	> 200	Before	mHealth	19 (54.2)	-0.48	0.632	mHealth	-2.98	0.003	
			Face-to-Face	21 (60.0)						
		After	mHealth	5 (14.2)	-0.32	0.744	Face-to-Face	-3.27	0.001	
			Face-to-Face	6 (17.1)						
**TG** **(mg/dl)**	> 150	Before	mHealth	23 (65.7)	-0.24	0.804	mHealth	-3.00	0.003	
			Face-to-Face	22 (62.8)						
		After	mHealth	14 (40.0)	-2.38	0.017	Face-to-Face	-0.70	0.480	
			Face-to-Face	24 (68.5)						
**AL** **(g/dl)**	< 3.5	Before	mHealth	8 (22.8)	0.00	1.00	mHealth	-2.33	0.020	
			Face-to-Face	8 (22.8)						
		After	mHealth	1 (2.8)	-0.58	0.558	Face-to-Face	-1.89	0.058	
			Face-to-Face	2 (5.7)						
**FER** **(µg/l)**	< 30 and > 300	Before	mHealth	23 (65.7)	-0.96	0.333	mHealth	-2.00	0.046	
			Face-to-Face	19 (54.2)						
		After	mHealth	15 (42.8)	-0.95	0.342	Face-to-Face	0.00	1.00	
			Face-to-Face	19 (54.2)						

SD: Standard Deviation, df; degree of freedom, IDWG: interdialytic weight gain, K: potassium, TC: total cholesterol, TG: triglyceride, AL: albumin, FER: ferritin

**Fig. 3:**
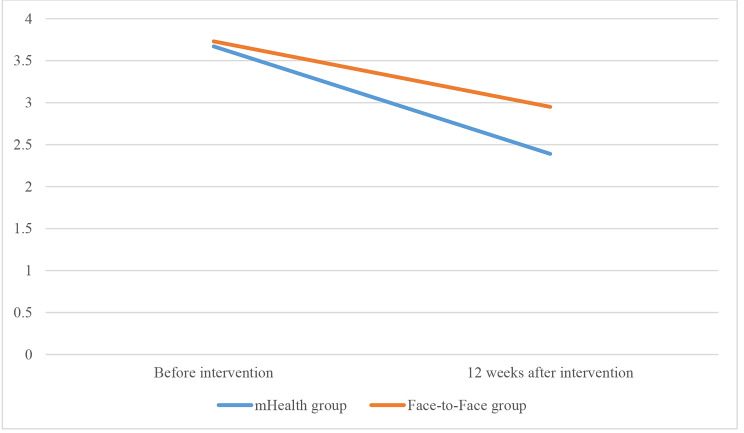
The IDWG changes before and after the intervention

​The paired-samples *t*-test showed that training with the Di Care app significantly reduced the mean IDWG (*p* < 0.0001), and the serum K (*p* = 0.001), P (*p* = 0.003), TC (*p* < 0.0001), TG (*p* < 0.0001), and FER (*p* = 0.038) levels, but the increasing trend in AL was not significant (*p* = 0.064) (Table 2). In the face-to-face training group, the mean IDWG in the patients (*p* < 0.0001), and the serum K (*p* < 0.0001), and TC (*p* < 0.0001) levels significantly decreased and AL (*p* < 0.0001) significantly increased; nevertheless, the serum K (*p* = 0.097), TG (*p* = 0.365), and FER (*p* = 0.162) levels did not significantly decrease before the intervention (Table 2; Fig. 3).

### Secondary outcomes

According to the criteria for poor treatment adherence based on the laboratory values ​​and IDWG mentioned in Table 3, the IDWG before the intervention was more than normal in 37.14% of the patients in the mHealth group and 60% of those included in the face-to-face training one. After training the patients with the Di Care app, their weight gain significantly diminished, so no one in this group had over 5.7% of their dry weight (*p* < 0.0001). In the face-to-face training group, 22.85% of the patients had gained weight more than normal after the intervention, which was statistically significant (*p* < 0.0001) (Table 3).

Of note, only 5.71% of the patients in the mHealth group had high serum K levels before the intervention, which reached zero after it (*p* = 0.157). In the face-to-face training group, none of the patients had a serum K level higher than 6 mmol/l before and after the intervention (Table 3). Since the serum P level in the patients before and after the intervention in both groups was not in the critical range, and they showed treatment adherence, it was not mentioned in the table.

The serum TC level before the intervention in 54.28% of the patients in the mHealth group and 60% of those in the face-to-face training one were not similar in the normal range. As the intervention was completed, this value decreased to 14.28% in the mHealth group (*p* = 0.003) and 17.14% in the face-to-face training one (*p* = 0.001), but this descending trend was not significantly different between the two study groups (*p* = 0.744) (Table 3).

​The serum TG level before the intervention in 65.71% of patients in the mHealth group and 62.85% of those in the face-to-face training one was not within the normal range, ​declining by 40% (*p* = 0.003) and 68.57% (*p* = 0.480), respectively, after the intervention. The results also demonstrated that training with the Di Care app could significantly moderate the serum TG level in the patients (*p* = 0.017) (Table 3).

​And the serum AL level in 22.85% of the patients in both groups was not in the normal range. After the intervention, the serum AL level in the group trained with the Di Care app reached 2.85% (*p* = 0.020) and that was 5.71% (*p* = 0.058) in the face-to-face training group. ​Training with the Di Care app lead to a significant growth in the serum AL level in the HD patients, although it was not significantly different compared with that in the face-to-face training group (*p* = 0.558) (Table 3).

​The serum FER level in the patients in the mHealth group (65.71%) and those in the face-to-face training group (54.28%) before the intervention were not within the normal range, decreasing to 42.84% after the intervention in the mHealth group (*p* = 0.046), but no changes were observed in the face-to-face training one (*p* = 1.00). However, the serum FER levels in both groups after the intervention were not significantly different (*p* = 0.342) (Table 3).

## Discussion

This study aimed to compare the effects of the mHealth app use and face-to-face training on the clinical and laboratory parameters of dietary and fluid intake adherence in HD patients. This study showed that the mean IDWG in 48.57% of the patients was higher than 5.7% of their dry weight. The rate of fluid intake non-adherence was differ from 33.6 to 68.8% in different studies [14, 30, 31]. The main reason for this discrepancy was the implementation of different measurement methods, such as questionnaires or weight measurements, at different times, to report fluid intake non-adherence in the given studies. ​As well, patients’ lifestyles could have affected the results. For example, the consumption of beverages, like tea, was very common among Iranian patients, which could lead to weight gain.

This study also indicated that the patients’ dietary adherence was appropriate in terms of the serum P level before the intervention. Considering K, only 2.85% of the patients had poor dietary adherence. In the previous studies, the mean serum K and P levels in the patients before the intervention had been in the normal range [18, 31]. In contrast, another study had shown that the serum K level in 16.4% of the patients and the serum P level in 30.2% had been higher than normal, and the patients had not shown dietary adherence [32]. ​As well, Ozen et al. reported abnormal serum P and K levels in 27.4% and 6.2% of the patients, respectively [14]. Such variations in the study results were attributed to the differences in the research settings and the patients’ eating habits. ​On the other hand, the patients could have more knowledge of K and P as well as foods containing them compared to other electrolytes and blood parameters, which could result in better control.

In the present study, the serum TC and TG levels respectively in 57.14% and 64.28% of the HD patients were higher than normal, and they had poor dietary adherence. Saini et al. found that the mean baseline serum TC and TG levels in the patients undergoing HD had been out of the normal range [33]. Soltani et al. also reported that the serum TG level was abnormal in 83% of the cases [34]. ​As well, the results of another study revealed that poor dietary adherence in HD patients could be associated with low serum K levels and high-fat profiles, particularly TC [16]. Considering the results of the present study and its comparison with previous researche, it was concluded that most HD patients had poor dietary adherence in terms of fat profiles that required some interventions in this regard.

In this study, the serum AL and FER levels were out of the normal range in 22.85% and 59.99% of patients, respectively, and they showed poor dietary adherence in this line. Significantly, the HD patients had self-administered ferritin-boosting medications. ​It seems that the main reason for the decrease in the mean serum FER level after the intervention in this study was the training provided regarding the side effects of taking such medications with no consultation. Similar results had been further reported, wherein the serum FER level in the patients had dropped after the administration of oral vitamin C supplements, and their need for erythropoietin-stimulating agents (ESAs) had dwindled [35]. The comparative study also found that 77% of American patients and 31% of European cases had poor dietary adherence due to AL deficiency [36]. In contrast with this study, results of another research showed that nutrition education did change FER and AL levels in comparison with the control group [20]. Of note, research on HD patients in terms of serum AL and FER levels was very limited, and no studies were found for comparison here.

​Both training methods did not significantly reduce the mean IDWG and the serum K, P, and TC levels in the patients. Clinically, the changes were more significant in the group trained with the Di Care app. Unlike face-to-face training, the Di Care app use reduced the serum TG level, increased AL, and modulated the serum FER level in the patients. Both methods promoted dietary and fluid intake adherence in the patients, which could significantly improve dietary and fluid intake adherence in the mHealth group.

In the present study, the mean IDWG and the serum TC and TG levels subsided in a significant manner in the patients trained with the Di Care app. The serum AL level also significantly dropped, but FER was adjusted. Despite the descending trend in the mean values of K and P, this decrease was not significant. Training HD patients by sending text messages in a study revealed that the mean serum P and K levels had diminished after the intervention but the IDWG had not significantly decreased [26]. A systematic review also demonstrated that the use of mobile apps by patients with CRF had improved some indicators, such as IDWG and serum K level, but further studies were required to evaluate such effects [37]. Another study examined the effects of researcher-made mobile apps and found that the method did not affect the serum K, P, FER, and AL levels in the patients [38]. Similar results had been further obtained in the study by Welch et al., using a self-monitoring program to assess nutritional status for six weeks, in which the program had not affected fluid intake and serum K and P levels [39]. The duration of the intervention in the study by Fakih was only one week, but that was one month here. In addition, unlike the Di Care app, the software implemented by Fakih only focused on dietary recommendations. In the study by Welch, the app recruited had no educational values, but simply monitored food and fluid intake in the patients.

This study showed a decrease in the mean serum K and P levels in the HD patients receiving face-to-face training, which was not statistically significant. These results agreed with the reports in Jahanpeyma et al. [18] and Hanifi et al. [40] studies, wherein face-to-face training and TFU reduced the serum K and P levels, and IDWG, but elevated AL. ​In contrast, another study had found that face-to-face training for one month had not affected P and K intake in the patients [41]. ​As well, the training sessions were twice a week for one hour, which was more than the recommended amount for the efficiency of training in these patients [42]. In the present study, face-to-face training was three sessions per week for about 10 min per session, which accounted for the differences in the results.

### Limitation

The minimum version of the Android operating system was not considered as inclusion criteria and it caused some problems in sampling. Moreover, the performance of the app was different in a few smartphone brands, models, and versions of the operating system. Therefore, some features of the app, especially medication reminders, did not work properly all the time while videos were playing without any problem. Furthermore, in order to watch videos, connecting to the internet was essential to download them from the server. Some of the patients couldn’t connect to the internet and some of them refused to participate since they did not want to use their smartphone data for this reason. Besides, despite of willingness of some patients to take part in the study, their families or other patients, who did not accept to participate in the study themselves, were prevented to enroll. Furthermore, it was hard for some patients to use the app, despite having all inclusion criteria, because of being old, not having the ability to work with their phone properly, etc. Furthermore, in the Di Care app, it was impossible for the patients to ask questions and receive answers, which was one limitation facing this study. During the face-to-face training, however, the patients could be in direct contact with the trainer. Due to their easy access to the Internet, many patients were likely to compare this training and the information found in cyberspace, so the information retrieved from unreliable sources sometimes contraindicated the training provided and could largely confuse the patients. Very limited studies have beenconducted on the effects of training methods, particularly mobile apps, on the blood parameters of HD patients, using questionnaires to evaluate the impact of interventions on various aspects of treatment adherence. In addition, the highest focus in the literature has been on the serum K, P, urea (UR), creatinine (CR), and Kt/v levels, but the effect ​of training methods on other values, ​​such as the serum AL, FER, TC, and TG levels has not been examined, which can minimize the possibility of comparing the study results. Therefore, it was suggested to shed light on such issues in future studies. Moreover, owing to the limitation of budget and time, it was preferred to give up measuring other variables like hemoglobin, sodium, and so on. Finally, due to the regulation of the center and other conditions, it was impossible to control ultrafiltration volume and sodium concentration. Almost all dialysis machines were of the same brand and model. However, there were some different ones and it was not possible to use the same machine for all patients. Regarding dialyzers, there was approximately no choice even for the center to provide different types of filters, especially high-flux dialyzers, due to a shortage of material in Iran and difficulties to provide them because of sanctions. As a result, the majority of them were PS 16 LF and PS 180 HF.

## Conclusions

The Di Care app use and the face-to-face training in the HD patients in the present study improved the clinical and laboratory parameters of dietary and fluid intake adherence. Both interventions reduced the IDWG, but they had diverse effects on the laboratory parameters. ​Both training methods decreased the mean serum K, P, TC, and TG levels, elevated AL, and adjusted the serum FER level. However, training with the Di Care app was more effective on several parameters compared to face-to-face training, and in similar cases, the mean reduction was greater in the mHealth group compared to the face-to-face training one. Due to the limited number of studies in this field, it was suggested to employ these interventions in future studies, considering a larger sample size in different groups. Given the widespread use of smartphones and the low costs of providing this type of software for a large group of patients as well as the positive effects of this type of intervention, they seem to be good alternatives to traditional training methods and other similar interventions.

## Data Availability

The datasets used and analyzed during the current study are available from the corresponding author upon reasonable request.

## Abbreviations

HD: hemodialysis
mHealth: mobile health
IDWG: interdialytic weight gain
K: potassium
P: phosphorus
TC: total cholesterol
TG: triglyceride
AL: albumin
FER: ferritin
CRF: chronic renal failure
GFR: glomerular filtration rate
ESKD: end stage of kidney disease
RRT: renal replacement therapy
SMS: short message service
TFU: telephone follow-up
IRCTs: Iranian Registry of Clinical Trials
SD: standard deviation
ESRD-AQ: end stage renal disease adherence questionnaire
ESAs: erythropoietin-stimulating agents
UR: urea
CR: creatinine
